# Strategies for the Identification and Assessment of Bacterial Strains with Specific Probiotic Traits

**DOI:** 10.3390/microorganisms10071389

**Published:** 2022-07-10

**Authors:** Edgar Torres-Maravilla, Diana Reyes-Pavón, Antonio Benítez-Cabello, Raquel González-Vázquez, Luis M. Ramírez-Chamorro, Philippe Langella, Luis G. Bermúdez-Humarán

**Affiliations:** 1 INRAE, AgroParisTech, Micalis Institute, Université Paris-Saclay, 78350 Jouy-en-Josas, France; edgar.torres-maravilla@inrae.fr (E.T.-M.); luis-maria.ramirez-chamorro@inrae.fr (L.M.R.-C.); philippe.langella@inrae.fr (P.L.); 2 Facultad de Medicina, Universidad Autónoma de Baja California, Mexicali 21000, Mexico; diana.reyes.pavon@uabc.edu.mx; 3 Food Biotechnology Department, Instituto de la Grasa (CSIC), Ctra. Utrera Km 1, Building 46, 41013 Seville, Spain; abenitez@ig.csic.es; 4 Laboratorio de Biotecnología, Departamento de Sistemas Biológicos, CONACYT-Universidad Autónoma Metropolitana Xochimilco, Mexico City 04960, Mexico; rgonzalezv@correo.xoc.uam.mx

**Keywords:** probiotics, strain-identification, isolation approach, in vitro screening

## Abstract

Early in the 1900s, it was proposed that health could be improved and senility delayed by manipulating gut microbiota with the host-friendly bacteria found in yogurt. Later, in 1990, the medical community reconsidered this idea and today probiotics represent a developed area of research with a billion-dollar global industry. As a result, in recent decades, increased attention has been paid to the isolation and characterization of novel probiotic bacteria from fermented foods and dairy products. Most of the identified probiotic strains belong to the lactic acid bacteria group and the genus *Bifidobacterium*. However, current molecular-based knowledge has allowed the identification and culture of obligatory anaerobic commensal bacteria from the human gut, such as *Akkermansia* spp. and *Faecalibacterium* spp., among other human symbionts. We are aware that the identification of new strains of these species does not guarantee their probiotic effects and that each effect must be proved through in vitro and in vivo preclinical studies before clinical trials (before even considering it as a probiotic strain). In most cases, the identification and characterization of new probiotic strain candidates may lack the appropriate set of in vitro experiments allowing the next assessment steps. Here, we address some innovative strategies reported in the literature as alternatives to classical characterization: (i) identification of alternatives using whole-metagenome shotgun sequencing, metabolomics, and multi-omics analysis; and (ii) probiotic characterization based on molecular effectors and/or traits to target specific diseases (i.e., inflammatory bowel diseases, colorectal cancer, allergies, among others).

## 1. Introduction

Bacteria have been known to humans since antiquity and have been regularly considered pathogens for crops, animals, and humans [1]. In recent years, the role of bacteria in the human intestinal ecosystem and their interaction with the host has been studied in depth [2], which has introduced a new field of research to elucidate the positive host-related effects of some groups of microorganisms with different implications and approaches. The probiotic concept evokes the “beneficial bacteria that, administered in adequate quantities may promote health benefits”, and this has lately promoted the generation of new functional preparations with more effects on specific conditions and outcomes [3].

The probiotic market has reached a new relevance in the agro-alimentary, nutritional, and even pharmaceutical economic sectors, with a constant increase in the number of studies in the field of probiotics [4,5]. Thus, there is interest in isolating novel probiotic candidates from different sources; research into both classical probiotic strains, such as lactic acid bacteria (LAB) and *Bifidobacterium* strains, as well as next-generation probiotics (NGPs) strains, is constantly growing. However, the evaluation of these strains should be performed with caution [4]. In general, classic probiotic characterization includes the isolation of bacterial colonies and the subsequent analysis of their morphology or motility, their resistance to the conditions of the gastrointestinal tract (GIT), their ability to adhere to intestinal cells, their production of antimicrobial compounds (i.e., such bacteriocins), their potential to reduce nitrate or other elements, and their antibiotic resistance [6]. Several probiotic bacteria can produce beneficial metabolites in the host, contributing to gut health by inhibiting pathogenic microorganisms [7]. However, since the latest observations from probiotic research advise that the health-promoting effects of one strain cannot be extrapolated to another, even of the same species, further studies are needed [3]. Presently, the challenge is to demonstrate the specific impact of each candidate strain in selected in vitro or in vivo models when targeting diseases. Some laboratories have established and proposed isolation and identification techniques; moreover, in vitro screening models are based on specific variables that could act as readouts for the correct selection of novel probiotic strains before being subjected to in vivo models prior to clinical models.

Probiotic research is increasing and the early stages in its production will provide the necessary information for current regulatory frameworks, which include safety, health benefit claims, and research. In this review, we summarize some attractive targets and readouts to demonstrate probiotic effects in vitro. Such findings could be key in the correct selection of novel probiotic candidates to treat human diseases, such as inflammatory bowel disease (IBD), irritable bowel syndrome (IBS), food allergies (FAs), and metabolic syndrome (MBS).

## 2. Isolation and Identification of Potential Probiotic Strains: Traditional and New Approaches

**Figure 1:**
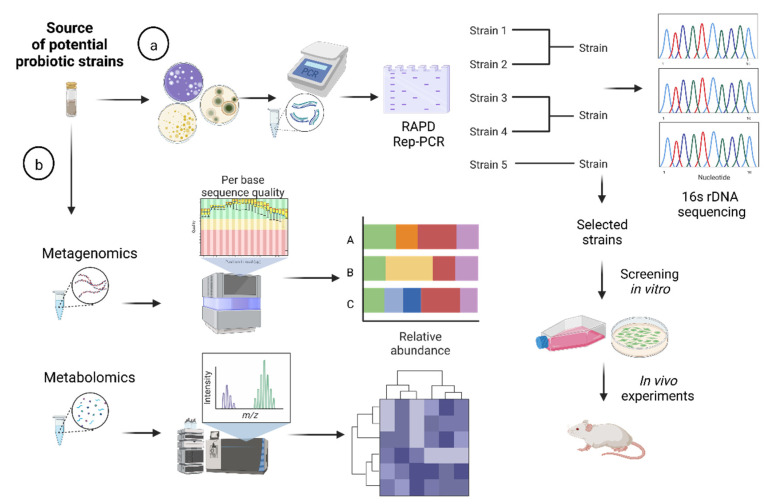
Representation of traditional and genomic-based approaches to identify potential probiotic candidates. (a) Classical isolation methods and further PCR genotyping (Rep-PCR, RAPD) allow strain identification, clustering, and selection of representative candidates for further in vitro and in vivo screening tests. (b) Metagenomics provides information on the relative abundance of microorganisms after massive sequencing of DNA (or cDNA), or RNA fragments from a sample and further bioinformatics analysis. Finally, metabolomics aims to identify, quantify, and characterize small-molecule metabolites produced by microorganisms from complex matrices. Two types of tools are required to analyze metabolomes: nuclear magnetic resonance and mass spectrometry. This figure was created with Biorender.com (accessed date: 9 June 2022).

Considerable efforts have been made in recent years to obtain potentially probiotic candidates from various animal or vegetable matrices. The techniques used for their selection and isolation have evolved considerably (Figure 1). Identifying nonconventional bacteria is necessary to elucidate their specific roles in various biological processes. Specifically, in the case of culture-dependent techniques, simple morphological characterization of these microorganisms is ineffective in documenting the complete diversity of a given profile [8]. In addition, new approaches/strategies are needed to identify and isolate novel bacteria of biological interest. In this context, amplification and sequencing of the 16S rRNA gene is the most widely used technique in bacterial phylogeny and taxonomy studies prior to further tests. However, the analysis of complex microbial community matrices presents several shortcomings: (i) the need to sequence many isolates, entailing significant cost and effort; (ii) the limitation of strain-level information; and (iii) the loss of taxonomic information due to the inherent limitations of culture-dependent techniques. While the first and second barriers can be solved by prior genotyping of isolates at the strain level using Rep-PCR or RAPD-PCR techniques and subsequent bioinformatics analyses of genetic profiles, the third can be solved by using culture-independent techniques (Figure 1a).

### 2.1. Repetitive Element Palindromic–Polymerase Chain Reaction (REP)

The Rep-PCR or repetitive element palindromic–polymerase chain reaction technique consists of PCR-amplified repetitive extragenic palindromic elements. It is a type of PCR based on primers designed to hybridize with repeated sequences interspersed in the genome, allowing different genetic profiles to be obtained between strains. The advantages of Rep-PCR include short analysis time and high discriminatory power while requiring a small amount of DNA, and it is an inexpensive procedure. Individual primers are commonly used, whereby enterobacterial repetitive intergenic consensus PCR (ERIC-PCR), extragenic repeating PCR ((BOX)-PCR), and (GTG)5-PCR sequences (5′-GTG GTG GTGGTGGTG-3′) are the most common [9]. However, a combined approach, which includes (GTG)5-PCR fingerprinting and AFLP, is more effective for LAB strain typing [10].

### 2.2. Random Amplified Polymorphic DNA (RAPD)

In the RAPD technique, or the random amplification of polymorphic DNA technique, unlike the previous one, the DNA fragments obtained are amplified from random genomic regions. This is because the sequences of the reaction primers are short but random, and the polymorphism observed between different strains consists of the presence or absence of randomly amplified DNA fragments. Similarly, when sequence information of newly isolated strains is not accessible, RAPD analysis can be first applied to develop strain-specific primers since the sequencing of the randomly amplified regions allows researchers to know their sequences [11].

Both the REP and RAPD techniques have proven useful for strain genotyping (Figure 1a). DNA fingerprints generated by either method are subsequently digitized and compared by bioinformatics analysis. One of the most widely used software is Bionumerics (bioMérieux, Applied Maths, Sint-Martens-Latem, Belgium). Bionumerics clusters DNA fingerprints based on different selected correlation parameters (Pearson product–moment correlation, cosine correlation, Dice, Jaccard, Jeffrey’s X, Ochiai, Okayama, Japan), and band numbers, creating DNA profiles that are finally clustered according to their similarities. Thus, the grouping of the strains into different clusters allows the selection of a single representative individual of each strain for subsequent identification and study of its potentially probiotic properties, representing a great deal of time and economic effort. For instance, Benítez-Cabello et al. [12] employed a GTG5-based Rep-PCR approach for genotyping 554 LAB isolates from table olive fermentation to study the probiotic potential of these strains subsequently. Bioinformatic analysis of the genetic profiles reduced the total genotypes to only 16 different strains, identified as *Lactobacillus pentosus* and *Lactiplantibacillus plantarum.* The *L. pentosus* strain, selected by LPG1 after the test as mentioned earlier, was then tested in an in vivo murine colitis model, showing a potent anti-inflammatory capacity and being classified as a potential probiotic strain [13]. GTG5-based Rep-PCR and further 16S rRNA gene sequencing techniques were also employed by Nami et al. [14] to identify 45 LAB (mainly, *Enterococcus*, *Lactobacillus*, and *Lactococcus* species) isolates from human vaginal microbiota with antibacterial effects. Finally, GTG5 rep-PCR and ERIC rep-PCR were employed by Guantario et al. [15] for strain genotyping of 19 *L. pentosus* and 7 *Loigolactobacillus coryniformis* potential probiotic candidates. Some strains showed good probiotic traits in vitro, and could exert pro-longevity and protective effects in the simplified model in *Caenorhabditis elegans*.

Recently, RAPD and further 16S rRNA gene partial sequencing approaches were used to identify the LAB strains responsible for the degradation of biogenic amines [16]. Different LAB strains, such as *Leuconostoc mesenteroides*, *Lactococcus lactis*, *Lacticaseibacillus casei*, *Lacticaseibacillus paracasei*, *Lacticaseibacillus* *rhamnosus*, and *Limosilactobacillus fermentum*; *Lentilactobacillus parabuchneri*, *L. paraplantarum* [17], *Streptococcus thermophilus* and *Streptococcus gallolyticus*, *Pediococcus pentosaceus*, *Enterococcus lactis*, and *Weissella paramesenteroides*, were identified [16]. Finally, RAPD, REP, and 16S rRNA gene sequencing have shown to be fast and cost-effective techniques with adequate discriminatory power and reproducible results for detecting and profiling probiotic bacterial candidates.

### 2.3. Omics Approaches for Screening Potentially Probiotic Candidate Strains

Omics science, which has emerged from the fast growth of genomic technology, has contributed substantially to recent advances in taxonomic studies by revealing the identity of the entire microbial population in a given environment. Genomic technology thus represents a key advantage over classical culture techniques when screening for potentially probiotic populations in a sample, the outcome of the first sequencing data being a sample of the metagenome, i.e., the collective genome of a microbial community. Metagenome sequencing represents not only an effort to identify different microbial populations, but also the first (and probably the best) opportunity to identify potentially interesting microbes (16S rDNA amplicon sequencing), their putative beneficial molecule pathways (whole-genome shotgun metagenomic sequencing, or WGS), and/or the beneficial molecules themselves (metabolomics) [18].

The metataxonomic approach is the most widely used approach for characterizing microbial communities’ composition, relative abundance, and evolution as a function of time or other clinical variables. This approach amplifies marker genes from total DNA extracted from a sample. For bacteria and archaea, variable regions (usually V3 and V4 of the 16S rRNA gene) are amplified with universal primers [19]. For fungi populations, the ITS1 region located inside the fungal nuclear ribosomal DNA (rDNA) with designed primers surrounding conserved regions, ITS1-F_KYO2 (18S SSU 1733–1753) and ITS2_KYO2 (5.8 2046–2029) 18S/ITS regions are commonly amplified [20,21], and the amplicons are massively sequenced. Subsequently, each sequence is assigned to a taxonomic group by mapping with those existing in public databases such as the Ribosomal Database Project (RDP) [22] or Greengenes [23] for bacteria and archaea, or UNITE [24] for fungi. Among the most widely used tools for bioinformatics analysis of metataxonomic sequences are QIIME [25], Mothur [26], MG-Rast [27], or R packages [28]. In all of them, the usual analysis steps include (a) quality control (identity ≥98%) and length (≥200 bp) of the sequences; (b) removal of chimeric sequences; (c) clustering of sequences by similarity and overlap; (d) taxonomic assignment; and (e) statistical analysis [29]. One of the challenges for metataxonomics is using DNA as a target molecule, which does not allow the identification of live or dead—active or inactive—microorganisms. A variant of the technique to solve this problem is the metataxonomic analysis of the active fraction. For this, RNA is extracted from the sample and converted into cDNA by reverse transcription providing information on active microorganisms. As a tool for screening probiotic candidates, its use has been varied. Recently, Kim et al. [30] led a randomized, double-blind, placebo-controlled, multicenter clinical trial to investigate the effect of probiotic capsules containing *Bifidobacterium bifidum* BGN4 and *Bifidobacterium longum* BORI in individuals aged >65 years over 3 months. The stool samples’ microbial load was examined by amplifying and sequencing the V3-V4 hypervariable regions of the 16S rRNA gene. They reported significant reductions in *Eubacterium*, *Clostridiales*, *Prevotellaceae*, and *Allisonella* abundances in the gut, resulting in favorable cognitive function outcomes and mental stress. Another metataxonomic study revealed increased abundances of short-chain fatty acids (SCFAs)-producing *Allobaculum* spp., *Coprococcus* spp., and *Bifidobacterium* spp in fecal samples after a 5-week oral administration regimen of *B. breve* CCFM1025 to chronically stressed C57BL/6J male mice [30].

### 2.4. Whole-Genome Shotgun Metagenomic Sequencing (WGS)

This approach, developed in the 1980s and 1990s, known as shotgun, is based on the massive sequencing of DNA (or cDNA) or RNA fragments without a previous amplification step. On the one hand, metagenome sequencing bypasses the critical bias introduced by the PCR process, since the fragments obtained are randomly selected from the total number of genomes present in the original sample [31] (Figure 1b). The set of all these fragments is considered representative of the set of bacterial genomes present in the original microbiota. On the other hand, this method is computationally more demanding, making the process more expensive. Tools for the characterization of metagenome sequences include GhostKOALA [32] and, more recently, PICRUSt2 [33]. The set of all these fragments is considered representative of the set of bacterial genomes present in the original microbiota. This technique is commonly used to identify probiotic species [34], and to determine the effects of probiotic consumption on the composition and function of gut microbiota [35]. Chen et al. [36] used shotgun metagenomics to study the effect of probiotic-induced microbiome modulation on intestinal inflammation. They reported that administration of *Lactiplantibacillus plantarum* LP-Onlly to Interleukin (*IL*)*-10^−/−^* mice, a murine model of IBD, induced proliferation of *Bacteroides* and *Akkermansia muciniphila*. These changes could correlate with an improved inflammation profile. Recently, Jangid et al. [37] employed shotgun metagenomic sequencing to reveal the prebiotic potential of a grain-based (GB) diet in mice. Taxonomic analysis of the sequenced reads revealed a significantly higher enrichment of probiotic lactobacilli in the GB group (a grain-based diet containing both soluble and insoluble fibers) compared with the control PIB group (purified-ingredients-based diet containing only insoluble fibers).

### 2.5. Metabolomics

This approach aims to identify and characterize small-molecule metabolites from complex matrices, thus providing a more direct strategy to study the potentially probiotic activity of a microbial community (Figure 1b). From an analytical point of view, metabolites’ qualitative and quantitative determination is considered one of the best markers of microbial activity. Since they are the end-product of a metabolic reaction, regardless of which microorganisms or enzymes participate in it, they can become a powerful tool for detecting probiotic microorganisms that produce compounds of biological interest in a given matrix. In the case of metabolic analysis of a sample such as feces, the study becomes much more complicated, since it not only contains the metabolic products of microorganisms and epithelial cells, but also receives a constant flow of substances with food intake. From an analytical chemistry point of view, two types of tools are required for metabolomes analysis: nuclear magnetic resonance and mass spectrometry. Furthermore, regardless of the technique used, there are two approaches in metabolomics: targeted (nature of metabolites known) and untargeted (nature of the metabolites unknown). The most-studied bacterial metabolites in feces are SCFAs, which originate from bacterial fermentation of complex dietary carbohydrates (fibers and starch). These fatty acids are of great importance in the physiology and nutrition of the gastrointestinal tract, presenting anti-inflammatory and anticancer properties, so they can be excellent markers of the probiotic activity of candidate microorganisms [38,39]. Metabolomic platforms are also used to investigate changes in neurotransmitters and hormones the concentrations linked to probiotics. Detection of changes aimed at more beneficial production of these molecules could be helpful for the selection of the best probiotic candidates. For example, intestinal tissues extracted from male C57B1/6J breastfed pups (stressed in early life by maternal separation), orally administered with *Bifidobacterium pseudocatenulatum*, were analyzed for monoamine neurotransmitters content by electrochemical detection (ECD) coupled with high-performance liquid chromatography (HPLC-ECD). Probiotic supplementation normalized dopamine and adrenaline concentrations. These effects were accompanied by improved stress and depression-like behaviors [40].

Calderón-Pérez et al. [41] performed a double metagenomics–metabolomics approach in a comprehensive gut microbiota analysis of 29 untreated hypertensive (HT) and 32 normotensive (NT) subjects. They first determined fecal microbiota composition by 16S rRNA gene sequencing and bacterial functions by metagenomic analysis. They subsequently examined microbial SCFAs in both plasma and feces, and trimethylamine N-oxide (TMAO) only in plasma. They found that the *Ruminococcaceae* NK4A214 strain, the *Ruminococcaceae* UCG-010 strain, the *Christensenellaceae* R-7 strain, the *Faecalibacterium prausnitzii*, and *Roseburia hominis* were significantly enriched in the NT group. In contrast, in HT patients, Bacteroides coprocola, Bacteroides plebeius, and genera of the Lachnospiraceae family were increased. They also reported that SCFAs showed antagonistic effects in plasma and feces, detecting significantly higher levels in feces and lower levels in plasma among HT subjects, indicating less efficient SCFA absorption. The synergy of culture-dependent molecular identification, whole-community metabarcode sequencing, and metabolomic approaches are more accurate in characterizing potential probiotic microorganisms. Similarly, analysis of multi-omics data from an integrated approach combining individual omics data, sequentially or simultaneously, would lead to a better understanding of the interactions of the molecules and microorganisms present, which would help assess the flow of information from one omics level to another and thus help bridge the gap from genotype to phenotype [42]. More recently, several studies have shown that combining omics datasets yields a better understanding and a clearer picture of the system under investigation [43]. Rasmussen et al. [44] combined complementary insights from multiple omics datasets of gut content samples in rainbow trout (Oncorhynchus mykiss) fed with a synbiotic feed supplement. They included bacterial 16S profiling, whole metagenomes, and untargeted metabolomics to investigate genomes assembled into bacterial metagenomes (MAGs) and their molecular interactions with the host’s metabolism. They revealed the following: (i) that feed supplements changed the microbiota and rainbow trout reared with feed additives had significantly reduced relative abundance of Candidatus Mycoplasma salmoninae in both the mid and distal gut content; (ii) metagenomics revealed that alterations in microbial arginine biosynthesis and terpenoid backbone synthesis pathways were directly associated with the presence of Candidatus Mycoplasma salmoninae; (iii) differences in gut microbiota composition between feed types were directly associated with significant changes in the metabolomic landscape, including lipids and lipid-like metabolites, amino acids, bile acids, and steroid-related metabolites. Thus, while the use of REP and RAPD and the clustering of their genetic profiles using bioinformatic techniques have been clearly shown to save time and effort when carrying out future characterization tests of probiotic microorganisms, omics techniques provide a global and broader view of the existing microbiota in an environment, in addition to possible interactions between molecules and microorganisms. Finally, probiotics are extensively used to modulate gut microbiota. Nevertheless, omics studies on the effects of probiotics are still relatively scarce. In this sense, Kiousi et al. [45] have recently compiled some of the applications of omics sciences performed in recent years to unravel the role of probiotics in health and disease. In the near future, the use of metagenomics and other omics sciences promises to expand our understanding of the mode of action of probiotics.

## 3. Target Screening to Select Bacterial Strains with Probiotic Properties

The impressive techniques for the identification and screening of different probiotics with beneficial therapeutic potential have been evolving along with the knowledge of each disease. In the following paragraphs, we try to gather all the potential readouts for the identification of essential probiotics with their in vivo application in IBD, IBS, food allergies (FAs), and metabolic syndrome (MBS)—four important pathologies of broad health interest.

### 3.1. Inflammatory Bowel Diseases (IBD) and Irritable Bowel Syndrome (IBS)

Although IBD and IBS are two different pathologies (disease versus syndrome) with multifactorial origins, they are characterized by gut microbiota dysbiosis and inflammation. IBD includes two main forms, Crohn’s disease (CD) and ulcerative colitis (UC), which are distinct, chronic, bowel-relapsing inflammatory disorders [46]. On the other hand, IBS is a functional bowel disorder and one of the most-diagnosed gastrointestinal illnesses. It is a symptom-based condition characterized by abdominal pain or discomfort, with altered bowel habits, in the absence of any other disease causing such symptoms [47].

During the inflammatory responses in IBD and IBS, extensive damage is produced in the intestinal junction’s proteins. This phenomenon compromises intestinal permeability, leading to the passage of different antigens, causing exacerbation of the inflammatory response, overproduction of reactive oxygen species (ROS), and proteolytic activity, among other types of damage [48]. It is known that modification of gut microbiota through the administration of probiotic strains can directly improve the pathogenesis of IBD and IBS [49,50]. Accordingly, the ability of a probiotic candidate bacteria to produce biofilms (and exopolysaccharides [EPS]) could be a good indicator, since the intestinal immune barrier is associated with biofilm and microbiota [51]. Another interesting experiment, which aims to determine whether a probiotic can reinforce the intestinal epithelial barrier, utilizes experiments in Caco-2 cells (or other colonic epithelial cells) using transfer-well plates, where trans-epithelial electric resistance (TEER) can be measured after challenges with a stressor—such as hydrogen peroxide (H_2_O_2_) or TNF-α—that decreases paracellular TEER values. In this way, it can be determined whether a probiotic candidate bacterium can protect cellular permeability while maintaining paracellular TEER values [52,53]. IBD and IBS are associated with an increase in proinflammatory cytokines, such as IL-1β, TNF-α, and IL-8, and an imbalanced IL-10/IL-12 ratio [54,55]; the importance of this status was previously demonstrated in IL-10^−/−^ mice [56]. Another key cytokine is IL-8, also known as CXCL8, a CXC-type chemokine originally identified as a leukocyte chemoattractant [57]. A model that attempted to mimic the inflamed colon conditions by coincubating TNF-α in HT-29 cells inducing IL-8 production by the NF-Κβ pathway was established, where a candidate bacterium can be considered as an anti-inflammatory if, after coincubation, it displays IL-8 blockage [58]. Coincubation with human peripheral blood mononuclear cells (PBMC) or mouse bone-marrow-derived dendritic cells (BMDCs) can also help to determine whether a bacterium induces the production of IL-10 (a cytokine with immunoregulatory properties) over that of IL-12 (a cytokine with proinflammatory properties) [59,60]. The importance of this balance was previously demonstrated in IL-10^−/−^ mice [56]. Finally, other cell types have been used to test the immunomodulatory properties of probiotic candidate strains, such as human and/or murine macrophages (THP-1 and RAW 264.7, respectively) [61,62,63].

Tian et al. [64] suggest oxidative stress as a target of IBD and ROS is a potentially pathogenic and critical factor in the onset, progression, and severity of these diseases. Probiotic bacterial strains could promote the development of some cellular antioxidant defense mechanisms and decrease the levels of proinflammatory cytokines and anti-apoptotic properties [65]. As demonstrated by Prete et al. [66] for *L. plantarum* O13 and C9O4 strains, these probiotics decreased ROS levels and IL-17F and IL-23 levels, making Th17 a critical pathway when trying to ameliorate chronic inflammation. In this context, some experiments testing the antioxidant properties of LAB have already been reported and could be used as screens. To determine the antioxidant activity of probiotics, this can be performed directly in intact cells and cell extracts of bacterial strains by 2,2-diphenyl-1-picrylhydrazyl (DPPH) free radical scavenging ability or by using in vitro cellular models [67]. For example, coincubation of *Lactobacillus* species in HT-29 cells in the presence of 2 mM H_2_O_2_, and subsequent measurement of thiobarbituric-acid-reactive substances, total antioxidant status (TAS), superoxide dismutase (SOD), glutathione peroxidase (GPx), catalase (CAT), and lipid peroxidation products [68]. Another example was proposed by Wang et al. [69], using the supernatants of *B. longum* CCFM752, *L. plantarum* CCFM1149, or *L. plantarum* CCFM10 in coincubation with A7R5 cells (a rat cell line that has been used to study the effect of angiotensin II on vascular oxidative stress). The results showed protection of the cells, inhibition of ROS production through antioxidant catalase enzyme, and NADPH oxidase activation. Another interesting strategy is using the cellular antioxidant activity (CAA) assay in Caco-2 cells, which consists of the simultaneous coincubation of bacteria and 2′,7′-dichlorofluorescein diacetate (DCFH-DA) at 37 °C/CO_2_. After 1 h of coincubation, cell damage was induced with 2,2′-azobis (2-methylpropionamidine) dihydrochloride (ABAP) generating a flux of peroxyl radicals, leading to the oxidation of DCFH into DCF, emitting a degree of fluorescence proportional to the level of oxidation [70].

In addition, anti-protease properties are another factor that are increasingly used as a therapeutic target for IBS. This is because of the high proteolytic activity observed in IBS patients [71]—the use of anti-proteases to counteract this activity takes relevance. Anti-protease of chemical origin [72] can be used, as well as bacteria that naturally expresses them. Motta et al. [73] demonstrated the efficacy of a recombinant *L. lactis* strain expressing human elafin anti-protease activity against inflammation in a mouse model of IBD, with similar protection properties to those of IL-10, also delivered by a recombinant *L. lactis* strain. Interestingly, some *Bifidobacterium* strains naturally produce a serine protease inhibitor called “serpin”. The work conducted by McCarville et al. [74] corroborated the benefits of a *Bifidobacterium* strain through serpin expression in preventing gluten-related immunopathology in a mouse model, which is characterized by a depletion of intestinal anti-proteases. Therefore, screening for strains with this feature could be an interesting approach.

Finally, enteric serotonin (5-HT) is responsible for gut motility, luminal secretion, visceral hypersensitivity, and inflammation. Dietary tryptophan can be converted into 5-hydroxytryptophan (5-HTP) by the tryptophan decarboxylase enzyme from gut bacteria, where 5-HTP is converted into 5-HT or tryptamine (a monoamine structurally similar to serotonin [5-HT]) [75,76]. Several species of the indigenous gut microbiota (*Bifidobacterium animalis* F1-7, *L. plantarum* FWDG, and *L. paracasei* F34-3 strains) have already been linked to the production of this mediator [77]. Ligands derived from tryptophan microbiota metabolism are endogenous activators of the Aryl hydrocarbon receptor (AhR, a conserved nuclear receptor). Evidence suggests that AhR activation suppresses IBD; in fact, some *Lactobacillus* strains can produce AhR ligands [78,79,80]. Therefore, AhR ligands production is another criterion when evaluating probiotic candidates in the context of IBD or IBS. 

### 3.2. Food Allergies (FA)

Homeostatic microbiota, together with the regulated activity of the immune system, allow the dominance of a tolerogenic microenvironment in organisms. Due to the large number of antigens that enter organisms through different sites (nose, mouth, skin, and others), a tolerogenic environment guarantees the generation of typical responses to harmless antigens. The diversity and richness of the gut microbiota enhance tolerogenic pathways to ingested antigens through the production of SCFAs, which can bind to G-protein-bound receptors on intestinal epithelia, triggering IL-18 production by the inflammasome and the release of antimicrobial peptides [81].

Dendritic cells (DCs) can drive the naïve T-lymphocyte towards a regulator profile and can contribute to its differentiation or induction when a microenvironment has the right conditions. However, when there are changes in the bacterial abundance or diversity, several tolerogenic factors (cytokines or SCFAs) decrease, favoring the pathogen, microorganism, or antigen entrance. In this scenario, activation of antigen-presenting immunological cells, such as DCs, in response to an inflammatory stimulus, can generate Th2 polarization and thus antigen sensitization with the consequent allergic reaction [82,83].

FA is a common food-related pathology in which tolerance to specific food antigens is reduced and atopic patients may be sensitized to different proteins in milk, wheat, eggs, soy, peanuts, and tree nuts, among others. To contribute to the patient’s quality of life, the use of probiotics and identification of novel strains that help in the different variables of allergy have been highly studied mainly implying positive effects through the modification of intestinal dysbiosis and the impact on microbial richness and diversity. They have also increased the tolerogenic-antigen-presenting cells to avoid aberrant and exaggerated immune responses and/or the regulatory T-cell population to maintain homeostasis, as well as decreasing the high permeability through the expression of tight junctions [84,85,86].

The production of tolerogenic DCs has been used primarily for the treatment of IBD and other diseases [87,88]. However, these techniques can also be explored in other intestinal-affecting pathologies such as FAs. As mentioned before, DCs may lead to the regulation of both innate and adaptive immune systems.

*Bifidobacterium* strains have been constantly reported to have regulatory effects on immune cell populations [89]. The in vivo models in which they have been tested include positive effects on the resolution of the FA clinical manifestations and a decrease in antigen-specific IgE titers and histamine secretion, and they deal with some aspects during their identification as positive strains for FAs. Konieczna et al. [90] worked on a model through the isolation of CD14^+^ human peripheral blood monocytes with the magnetic-activated cell-sorting system (MACS) and then stimulated for 5 days to generate monocyte-derived dendritic cells, which were later stimulated with carboxyfluorescein succinimidyl ester (CFSE)-stained *Bifidobacterium infantis* 35624. Several features, such as binding and internalization of the probiotic, as well as expression, CD80 and CD86, secretion of IL-10, and even induction of autologous FoxP3 T regulatory cells, were important results of the work. Fu et al. [91] showed that the use of the *B. infantis* 14.518 strain in allergic Balb/c mice suppressed the FA responses and significantly increased the tolerogenic CD103^+^ DCs population, which also induced T regulatory cells on mesenteric lymph nodes (MLNs) and modified the indigenous microbiota. Other models, such as the one proposed by Santos et al. [92] in BALB/c mice with therapeutic administration of *B. longum* 51A, highlight the importance of TEER and immunomodulation in identification techniques to assess the decreased permeability and changes in the levels of inflammatory cytokines such as IL-4, IL-5, IL-6, IL-13, and TNF-α.

Although the generation of regulatory DCs and T cells through the administration of probiotics has been analyzed as a fundamental part of the identification of their properties for a long time, the results obtained in vivo have continued to evolve in recent years. In 2010, Kwon et al. [93] reported the use of a probiotic mixture containing *L. acidophilus*, *L. casei*, *Limosilactobacillus* *reuteri*, *Bifidobacterium bifidium*, and *Streptococcus thermophilus* administrated to BALB/c mice. Then, authors performed an analysis of the cellular characteristics of the different cell populations by isolating CD11c^+^ DCs from MLN and then cultured them with splenic CD4^+^ T cells from transgenic or wild type BALB/c mice labeled with 10 mM CFSE during the differentiation assay. Finally, they tested an adoptive transfer assay on atopic-dermatitis-induced mice. The probiotics caused the generation of CD4^+^FoxP3^+^Treg lymphocytes and ameliorated the suppressor effects of CD4^+^CD25^+^ nTregs. In other models, increased CD4^+^Foxp3^+^T-cell population in MLNs has been assessed alone (Hee-Soon et al. [94] or indirectly through the increase in mucosal CD103^+^ dendritic cells in Peyer’s patches and MLNs that can convert naïve T cells to FoxP3+ T regulatory cells (Ma et al. [95] on BALB/c models of ovalbumin FA).

The production of several microbiota metabolites such as SCFAs, required for T-cell induction and metabolism [96,97] and the generation of tolerogenic cytokines [98], are also thought to be necessary for the identification of novel probiotics for FA. It is now known that these processes are also linked with GPR43 [99] through the activation of mTOR and STAT3 and epigenetic modifications via inhibition of histone deacetylases, and through the antigen response by marginal-zone B-cell lymphocytes [100]. STAT 3 is known to enhance allergic reactions [101] through a significant decrease in cytokines and chemical-released mediators assessed in murine and human mast cells. Other molecules, such as indoleacrylic acid, have been studied as a part of the positive effect of bacteria in restoring intestinal tryptophan metabolism and thus mitigating inflammatory responses [102]. Lu et al. [103] tested the combination of a mixture with *L. plantarum* CCFM1189, *L. reuteri* CCFM1190, and *Bifidobacterium longum* CCFM1029, and all showed a decrease in antigen-specific IgE, histamine, and clinical manifestations. Some microbiota changes—such as an *Akkermansia* increase linked to *L. plantarum* or increased indoleacrylic acid in fecal samples generated by *L. plantarum* and *L. reuteri*—are important findings of the work.

Specific in vitro effects may help identify beneficial probiotics involved in allergy, including the blockage of genes related to high-affinity IgE receptor subunits α and γ or histamine H4 receptor. This has been described for *L. rhamnosus* LGG and Lc705 on mast cells in vitro assays [104], or through the suppression of the allergic response via the selective apoptosis of the mast cells by using extracellular vesicles, as has been seen for *Bifidobacterium longum* KACC 91563 [105], but not fully explored for other probiotics. Oxidative stress is another potent inducer of allergic inflammation, and some probiotics have even induced a therapeutic effect by mediating it. *B. infantis* was also tested on an ovalbumin BALB/c FA model in 2018 [106], where it attenuated the antigen-dependent antibody responses and pathology manifestations; however, it also mediated protection via its superoxide dismutase and decreased intestinal malondialdehyde. Furthermore, in in vitro assays, it inhibited ROS production from DCs and decreased TIM4 expression by deactivating STAT6.

Finally, since increased permeability is an important parameter manifested during FAs, the factors affecting it should be considered to find the best strains for the pathology. Microbiota changes and mast cell protease effects are important factors involved in this variable. Using Caco-2 monolayers, Feng et al. [107] analyzed exposure to acetate, propionate, and butyrate (the main SCFAs) with or without stimulation by lipopolysaccharide (LPS). TEER and paracellular permeability were measured with a Millicell-ERS voltohmmeter and fluorescein-isothiocyanate-labeled dextran. The study identified that the SCFAs inhibited LPS-induced NLRP3 inflammasome activation, acting as energy substances to protect intestinal barrier function in Caco-2 cells.

Tulyeu et al. [108] worked on a Brown Norway SPF male rat model of ovalbumin FA using *Clostridium butyricum* and *Limosilactobacillus reuteri* assessing histological changes in the intestinal tissue and permeability changes through the upregulation of the expression of *ZO-1*, *occludin* and *claudins* 1, 3, 5, 7, 8, 9, and 15. After that, Jin et al. [109] designed a model with Sprague Dawley rats delivered by cesarean section. After sensitization to ovalbumin, they exhibited intestinal permeability and intestinal barrier impairment through decreased expression of *ZO-1*, *occludin*, and *claudin-1*, and had a strong allergic response to the food challenge. The authors identified a probiotic mixture with *Bifidobacterium longum* subsp. *infantis*, *Lactobacillus acidophilus*, *Enterococcus faecalis*, and *Bacillus cereus* and evaluated an increased level of tight junction proteins which also attenuated the manifestations, antibodies, and histamine response—critical for FAs.

### 3.3. Metabolic Syndrome (MBS)

Metabolic syndrome (MBS) refers to the presence of a set of specific risk factors for cardiovascular disease: a combination of insulin resistance (or diabetes), high blood pressure (hypertension), and obesity [110]. The latter condition is characterized by a chronic, low-grade systemic inflammatory disease that is complex and multifactorial, with genetic and environmental factors involved [111]. The proinflammatory response is partially generated in adipose tissue; interestingly, probiotics have also been proposed to suppress chronic inflammation in murine models by modulating adipokines secretion in the adipose tissue and by upregulation of adiponectin in adipocytes [112]. In addition, obesity is characterized by intestinal dysbiosis, which contributes to the increase in proinflammatory cytokines. Therefore, immunoregulatory or anti-inflammatory properties (as before mentioned for IBD, IBS, and FA) are probiotic effects to evaluate in candidate strains. Fabersani et al. [113] proposed an appropriate system to select strains with the ability to modulate adipokine secretion; based on coincubation of bacterial candidates and macrophages stimulated with *E. coli*–LPS (serotype O26:B6), with the following readouts expression in macrophages: TNF-α, IL-6, IL-10, monocyte chemoattractant protein-1, leptin, and the leptin receptor (Ob-Rb). Another factor contributing to the low-grade inflammatory state in obesity is gut dysbiosis: previous studies have shown a decrease in the number of bacterial populations (i.e., LAB, *Bifidobacterium* spp. and *Akkermansia* spp.). In this regard, probiotics could be used to restore gut microbiota imbalance [114].

One of the modifications during dysbiosis is through the SCFAs levels, which also have an essential role in obesity, since these fatty acids also work as signaling molecules, capable of activating some G-protein-coupled receptors. GPR43 (free fatty acid receptor 2) is known to regulate energy balance in the host through the gastrointestinal and adipose tissues, and is being explored as a potential therapeutic alternative for metabolic diseases [115,116]. Increased production of acetate, propionate, and butyrate (SCFAs) as a possible consequence of microbiota changes has been associated with modulation levels of ghrelin, insulin [117,118,119], GLP-1 [120], leptin [121], peptide YY [122], and other regulating hormones for satiety and metabolism. Evidence supports the role of SCFAs in improving weight gain (propionate and butyrate), food intake, and glucose homeostasis (acetate)—effects that may result from the action of SCFAs at receptors on eukaryotic cells. Studies have shown that acetate can suppress insulin signaling in adipocytes, inhibiting fat accumulation in adipose tissue. Hence, SCFAs act at different levels by decreasing the inflammatory state that reduces insulin resistance, increasing the protective glucagon-like peptide-1 (GLP-1) secretion that stimulates insulin release and improving β-cell function [123]. Thus, an important target to evaluate in probiotics is the ability to simultaneously produce the previously described SCFAs, which can be determined in vitro by gas chromatography [124].

Hypercholesterolemia is another factor that induces adipose dysfunction in obesity. Several probiotics have been reported to have a hypocholesterolemic effect in animal models due to reductions in lipid and cholesterol levels, with several proposed mechanisms of action [125]. One of these is the activity of bile salt hydrolase (BSH): in the gastrointestinal tract it can deconjugate bile salts, inducing the de novo synthesis of conjugated salts at the expense of cholesterol, resulting in decreasing serum levels and alterations in energy homeostasis, thus making BSH a clinically significant enzyme [126]. This activity can be tested by a simple plate test and quantified by spectrophotometry or quantitative high-performance thin-layer chromatography (HPTL) [127,128]. *Lactobacillus plantarum* DGIA1, a potentially probiotic strain isolated from a double cream cheese from Chiapas, Mexico, showed excellent deconjugation activities in sodium glycocholate, glycodeoxycholate, taurocholate, and taurodeoxycholate. Additionally, the commercial probiotic yeast *Saccharomyces boulardii* showed deconjugation activities against sodium glycodeoxycholate, taurocholate, and taurodeoxycholate (100%, 57%, and 63%, respectively) and a weak deconjugative activity (5%) in the case of sodium glycocholate [128]. Using a ninhydrin assay, *Lactobacillus casei* J57 isolated from pulque (a Mexican fermented beverage) showed the ability to deconjugate primary and secondary bile salts as follows: 44.91, 671.72, 45.27, and 61.57 U/mg, to glycocholate, taurocholate, glycodeoxycholate, and taurodeoxycholate, respectively [127].

Another mechanism related to cholesterol decrease is bacteria’s cholesterol assimilation, which lowers the luminal cholesterol levels which are available for absorption. Some bacteria from *Lactobacillus* genus can produce ferulic acid by ferulic acid esterase (FAE), which can inhibit hepatic 3-hidroxi-3-metilglutaril-coenzyme A (HMG-CoA) reductase and reduce hypercholesterolemia and hyperglycemia [125]. Ferulic acid is a phenolic acid that presents a wide range of potential therapeutic effects useful in the treatments of cancer, diabetes, lung and cardiovascular diseases, as well as hepatic-, neuro-, and photoprotective effects and antimicrobial and anti-inflammatory activities. Its potential can be attributed to its ability to scavenge free radicals [129]. It is worth mentioning that BSH activity and FAE can be determined in vitro by east plate assays [127,130]. Finally, with obesity being important in MBS induction as a chronic low-grade pathology with an increase in oxidative stress, several probiotics with antioxidant features have also been tried.

The in vitro cholesterol-lowering abilities of the following strains, isolated from healthy infants, were tested by Zhang et al. [131]: *Enterococcus faecium* strains WEFA23, WEFA24, WEFA26, WEFA28, WEFA30, and WEFA32; *Enterococcus hirae* strains WEHI01 and WEHI02; *Enterococcus durans* strain WEDU02; *Enterococcus casseliflavus* strain WECA01. The WEFA23 strain of *E. faecium* was the one with the best cholesterol removal and adhesion ability, potentially ameliorating obesity and hyperlipemia in an in vivo high-fat-diet rat model.

Another desired property in the MBS context is improving mitochondrial biogenesis and other intracellular dynamics to improve the obesity state. The antioxidant effects of probiotics for metabolic syndrome have also been highlighted in vivo models. Huo et al. [132] described the efficacity of *B. animalis* subsp. *lactis* A6 to increase eNOS, Pgc-1 α, *Nrf-1*, estrogen-related receptor α, β-hydroxyacyl CoA dehydrogenase, and uncoupling protein-1 and mRNA and protein expression in a mice model of C57BL/6 males; moreover, the probiotic candidate strain accelerated weight and fat mass loss, changing the microbiota and thus decreasing serum LPS and proinflammatory cytokines such as TNF-α.

Martorell et al. [133] verified the reduction in body fat and triglycerides and the antioxidant effect of *B. animalis subsp. lactis* CECT 8145 in an innovative model in *C. elegans* strain N2 (wild type), and 13 of its mutant strains. *B. animalis* protected the nematode from the oxidative stress of H_2_O_2_ in a 5 h experiment, assessing anti-inflammatory activity in the nematode and fat and triglyceride reduction. Carreras et al. [134] continued exploring the same strain on a model of 5-week-old Zücker fatty rats, assessing a decrease in the total cholesterol/HDL cholesterol, plasmatic glucose, insulin ratio, ghrelin, and an antioxidant response through a decrease in malondialdehyde levels.

## 4. Discussion and Conclusions

**Figure 2:**
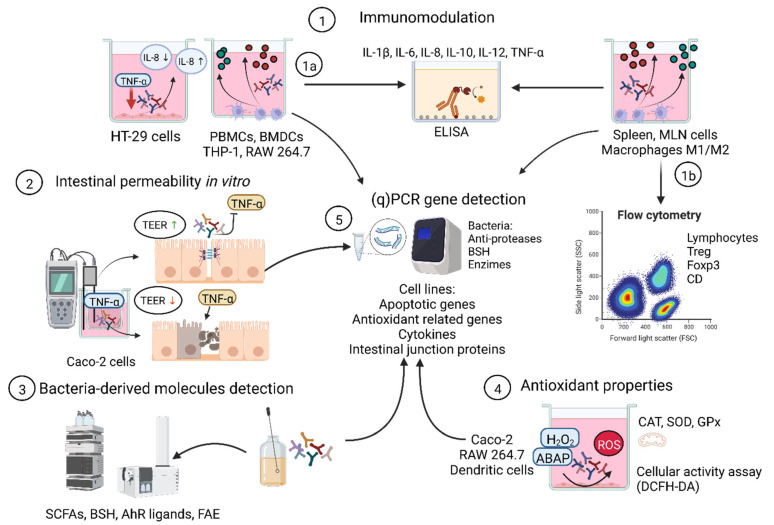
Outline of in vitro assays for screening probiotic candidate strains in three different settings: inflammatory bowel diseases and irritable bowel syndrome; food allergies; and metabolic syndrome. Probiotic candidate strains can be screened with different approaches, for example: (1) immunomodulatory properties can be screened by quantification of key cytokines through coincubation in different cellular models; supernatant coincubation can be determined by (1a) enzyme-linked immunosorbent assay (ELISA); cells subpopulations (activation and differentiation) can be determined by (1b) flow cytometry; (2) probiotic candidate strains can be screened by their ability to reinforce the intestinal barrier, and by measuring the para-cellular trans-epithelial electric resistance values of human colon cell lines; (3) probiotic candidate strains can be screened by identification of potential bacteria-derived molecules with probiotic properties; (4) probiotic candidate strains can be screened by their antioxidant properties from intact bacterial cells by chemical test or by using cellular models; (5) finally, all the probiotic properties mentioned here can be studied by gene expression using quantitative PCR, directly from bacteria cells and/or cell lines after interacting with the strains. PBMC—human peripheral blood mononuclear cells; BMDCs—mouse bone-marrow-derived dendritic cells; MLN—mesenteric lymph nodes; Macrophages M1—classically activated macrophages; M2—alternatively activated macrophages; TEER—trans-epithelial electric resistance; SCFAs—short-chain fatty acids; BSH—bile salt hydrolase; AhR—aryl hydrocarbon receptor ligan; FAE—ferulic acid esterase; ROS—reactive oxygen species; ABAP—2,2′-azobis (2-methylpropionamidine) dihydrochloride; CAT—catalase; SOD—superoxide dismutase; GPx—glutathione peroxidase; DCFH-DA—2′,7′-dichlorofluorescein diacetate. This figure was created with Biorender.com (accessed date: 30 May 2022).

Currently, there is an increased demand for health-promoting products and, consequently, there is a continuous, rapid development of new products. Due to the latest insights into human and animal gut microbiota data, probiotic research has been undergoing exponential growth over recent decades [135]. In this review, we cite effective alternatives for identifying potential probiotic strains (both culture-dependent and -independent methods, while addressing the limitations to date), and in vitro or in vivo experiments with different approaches to screening probiotic strains in three distinct model diseases (Figure 2).

Evolving from limited culture-based techniques, the combined knowledge of 16S rDNA, metagenomics, and metabolomics allows a better elucidation of probiotic colonization and direct or microbiome-mediated impacts (or their absence) on the human host, paving the way for the improved efficacy and safety of probiotics. Firstly, the evaluation of matrices and/or sources of probiotic strains (i.e., traditionally fermented and dairy food products, or those of human origin) must be assessed. Here, we showed a pipeline of technological developments, including massively parallel DNA and RNA sequencing, metabolomics, and other omics that have notably boosted the field of microbiome research in recent years. These methodologies can be leveraged to improve our deep mechanistic understanding of the basic concepts related to probiotic consumption, including their interaction rules with the indigenous microbiome and impacts on human host health. These new approaches have increasingly displaced the use of classical and culture-dependent identification methods. Although metataxonomic and metagenomic studies do not imply the isolation of the microorganism, they provide us with generalized knowledge of the total existing microbiota without prior sequence information. This knowledge allows us to design the best strategy for creating culture- and media-specific requirements for the isolation of the microorganism of interest [136,137]. Thus, the importance of using mixed approaches including culture-independent and culture-dependent techniques for detecting and subsequently isolating potentially probiotic microorganisms is highlighted. In addition, comparing complex samples (such as those of human origin) has been used to isolate beneficial bacterial strains. For example, Sokol et al. [138] observed a decrease in the abundance and biodiversity of intestinal bacteria in Crohn’s disease (CD), particularly a major member of Firmicutes, *F. prausnitzii*. A similar strategy was conducted to identify *Christensenella minuta* in patients without IBS [139].

As summarized above, the emerging technologies for the identification and screening of probiotic strains with therapeutic potential are extensive. Such technologies have also started to grow in the neurological, psychiatric [140], oncologic [141], and other health-related fields. This fact has expanded the use and applications of beneficial bacteria with promising results about their potential to restore human health by maintaining or restoring intestinal and systemic homeostasis. The specific cause of inflammation is one of the most-analyzed and still-unsolved puzzles of modern medicine; even so, several critical probiotic mechanisms have been identified for successful therapy of intestinal inflammation in IBS, IBD, FAs, and MBS. It is important to note that, even though there are many shared points during the identification of the potential probiotic activity—such as antioxidant effects, changes in regulatory lymphocyte populations, decrease in inflammatory cytokines, or even the ability to generate several substances during their metabolic pathways such as SCFAs—the complexity of the pathophysiological changes and the gut microbial ecosystem are essential factors that could restrict the speed of this progress. The ability of bacteria to restore and/or counteract the exacerbated inflammatory response is an attribute to be considered in searching for functional and safe probiotics with therapeutic effects. We know that the proinflammatory response is a complex process and should not be minimized to only one or few cytokines. Still, at least in this context, the described in vitro cellular experiments could help in the proper selection of probiotic candidates. Not only classical probiotic candidate strains (i.e., *Lactobacillus, Bifidobacterium* species), but also the NGPs, such as *F. praustnitzii* [142] and *C. minuta* [143], have been tested in this cellular model.

Several novel targets, such as probiotics which produce retinoic acid, an important metabolite for the generation of T-regs [144], the increased production of antioxidants such as astaxanthin [145], or even the quorum-sensing capacities of the microorganism [146], will be crucial parts of the probiotic screening for their application in different models and pathologies. In addition, the newest identification and characterization techniques are evolving so fast that they include organoid culture, spheroids [141], big data, and machine learning, which will soon be everyday tools for probiotics identification [147].

Current knowledge of probiotics regarding their identification in different matrices, their mechanisms, and their effects will continue to be explored. The more specific the strain screening and identification techniques are, the wiser the choices for probiotic use and recommendation will be. As it continues growing, the probiotics field remains promising. However, many of their effects are still only half understood and, as all probiotic strains are unique, several strategies must be developed in the future to understand each of them fully.
